# Protective Vaccination against Papillomavirus-Induced Skin Tumors under Immunocompetent and Immunosuppressive Conditions: A Preclinical Study Using a Natural Outbred Animal Model

**DOI:** 10.1371/journal.ppat.1003924

**Published:** 2014-02-20

**Authors:** Sabrina E. Vinzón, Ilona Braspenning-Wesch, Martin Müller, Edward K. Geissler, Ingo Nindl, Hermann-Josef Gröne, Kai Schäfer, Frank Rösl

**Affiliations:** 1 Division of Viral Transformation Mechanisms, German Cancer Research Center (DKFZ), Heidelberg, Germany; 2 Research Group Tumorvirus-specific Vaccination, German Cancer Research Center (DKFZ), Heidelberg, Germany; 3 Department of Surgery, Experimental Surgery Division, University of Regensburg, Regensburg, Germany; 4 Department of Dermatology, Venereology and Allergy, Skin Cancer Center Charité, University Hospital of Berlin, Berlin, Germany; 5 Department of Cellular and Molecular Pathology, German Cancer Research Center (DKFZ), Heidelberg, Germany; National Institute of Allergy and Infectious Diseases, United States of America

## Abstract

Certain cutaneous human papillomaviruses (HPVs), which are ubiquitous and acquired early during childhood, can cause a variety of skin tumors and are likely involved in the development of non-melanoma skin cancer, especially in immunosuppressed patients. Hence, the burden of these clinical manifestations demands for a prophylactic approach. To evaluate whether protective efficacy of a vaccine is potentially translatable to patients, we used the rodent *Mastomys coucha* that is naturally infected with *Mastomys natalensis papillomavirus* (MnPV). This skin type papillomavirus induces not only benign skin tumours, such as papillomas and keratoacanthomas, but also squamous cell carcinomas, thereby allowing a straightforward read-out for successful vaccination in a small immunocompetent laboratory animal. Here, we examined the efficacy of a virus-like particle (VLP)-based vaccine on either previously or newly established infections. VLPs raise a strong and long-lasting neutralizing antibody response that confers protection even under systemic long-term cyclosporine A treatment. Remarkably, the vaccine completely prevents the appearance of benign as well as malignant skin tumors. Protection involves the maintenance of a low viral load in the skin by an antibody-dependent prevention of virus spread. Our results provide first evidence that VLPs elicit an effective immune response in the skin under immunocompetent and immunosuppressed conditions in an outbred animal model, irrespective of the infection status at the time of vaccination. These findings provide the basis for the clinical development of potent vaccination strategies against cutaneous HPV infections and HPV-induced tumors, especially in patients awaiting organ transplantation.

## Author Summary

Organ transplant recipients (OTR) frequently suffer from fulminant warts that are induced by cutaneous human papillomaviruses (HPV). Moreover, some skin HPV types may also be involved in the development of non-melanoma skin cancer. Mimicking the situation of immunosuppressed OTR who acquire cutaneous HPV infections already in childhood, we explored the efficacy of a vaccine in infected animals that additionally underwent immunosuppression. We demonstrate for the first time the success of a vaccine against a skin papillomavirus in a natural outbred animal system, which completely prevents both benign and malignant skin tumor formation even under immunosuppressed conditions. Hence, our study provides the basis for clinical development of a vaccine against cutaneous HPV infections, which may be particularly useful in transplant recipients.

## Introduction

Papillomaviruses (PVs) infect mucosal and cutaneous squamous epithelia, where they can cause hyperproliferative lesions. In the case of high-risk genital human papillomavirus (HPV) types, a causative link has been established between HPV infection and the development of malignant diseases, especially cervical carcinoma [1]. For cutaneous types, the association between HPV infection and skin cancer is still a matter of debate [2], although there is increasing evidence that supports their role as a cofactor with UV radiation in the development of non-melanoma skin cancer (NMSC) [3]. Indeed, it has been shown that certain cutaneous HPVs display transforming properties and tumorigenic features, both *in vitro* and *in vivo* [4]–[7]. Furthermore, epidemiological data that support an association of HPV infection and NMSC have been found for two distinct populations: patients with the rare hereditary disease *Epidermodysplasia verruciformis* (EV) and immunosuppressed organ transplant recipients (OTR). Compared to the general population, the incidence of NMSC is up to 250-fold higher in OTR [8], [9]. Additionally, OTR suffer from benign and premalignant skin lesions, such as actinic keratosis, keratoacanthomas and cutaneous warts, which are indisputably caused by cutaneous HPVs [10], [11]. Such lesions appear over a large area of the skin, persist for years and significantly reduce life quality. Hence, the high incidence of PV-induced warts and premalignant lesions in immunosuppressed OTR represents a great burden, which demands new prophylactic strategies to prevent such skin manifestations. We suggest that peri-transplant immunization with vaccine against cutaneous HPV types could reduce the incidence of virus-induced skin lesions that can progress to NMSC.

Vaccination against genital HPV types is currently being used worldwide to prevent infection and, in turn, the development of PV-induced lesions in the mucosa, including cervical carcinoma. The two licensed vaccines are composed of HPV virus-like particles (VLPs), which elicit high titers of neutralizing antibodies that protect from a subsequent infection by the targeted HPV types [12]. Both vaccines are very effective when applied in individuals with no previous exposure, but efficacy decreases when analyzed in patients with positive HPV serology [13].

A unique preclinical model to investigate PV-associated skin tumorigenesis is the African multimammate mouse *Mastomys coucha*, originally taxonomically designated as *Mastomys natalensis* [14]. This species belongs to the rodent family Muridae, as the laboratory mouse *Mus musculus*. The colony maintained at the German Cancer Research Center (DKFZ) is naturally and persistently infected with *Mastomys natalensis papillomavirus* (MnPV) and *Mastomys coucha papillomavirus 2* (McPV2), which - like cutaneous and genital HPVs - infect epidermal and mucosal tissues, respectively [15], [16]. Throughout their lifetime these animals spontaneously develop epithelial lesions of the skin (mainly papillomas and keratoacanthomas) as well as papillomas at the tongue and condylomata at the anus, vulva and penis. Both papillomaviruses persist episomally without any indication of integration, analogous to cutaneous HPVs [16], [17]. Naturally MnPV-induced lesions rarely regress and can efficiently form squamous cell carcinomas (SCC) after a single topical application of a carcinogen followed by repeated challenge with a tumor promoter [18]. Additionally, the potential oncogenic capacity of MnPV could be also demonstrated in transgenic mice carrying the E6 oncoprotein, in which viral expression was targeted to the basal layer of the skin [19]. In contrast to the cottontail rabbit papillomavirus [20], MnPV shares the trademark of cutaneous HPVs that lack a functional E5 open reading frame (ORF) [21]. This fact, together with the alignment of *cis*-responsive elements in its regulatory region and the phylogenetic assessment of parts of the E6, E1, and L1 ORFs, indicates that MnPV is related to HPV types found in lesions of cutaneous epithelia, in particular to those associated with EV [22].

Considering the natural infection mode, *Mastomys* acquire the virus very early after birth, as MnPV-DNA is found in the skin of four-week-old animals at the same time as seropositivity against viral antigens [23]. In fact, the infection status within our animal colony mimics the situation of some cutaneous HPVs, which are also acquired early during childhood [24], [25]. As previously shown in a large serological study, a strong correlation between MnPV L1-specific antibodies and benign skin tumor formation is discernible. Notably, among the early expressed viral proteins, strong antibody responses against MnPV E2 can be measured at an age of one month. A prospective study additionally revealed that E2 seropositivity marks early and late stages of infection. Together with high anti-L1 reactivities at an age of 4.5 months, subsequent tumor development could be predicted [23]. In contrast to other animal models [26], [27], all progression stages can be monitored in an immunocompetent laboratory animal, starting from primary infection in newborns until lesion development in a natural host [28]. Since inbred strains may vary substantially from outbred counterparts in their immune response [29], the outbred character of these animals does not only mimic the genetic heterogeneity in humans, but also allows to determine whether the protective effect of a VLP vaccine is representative and transferable to patients. Hence, the success of a VLP-based vaccination can be readily monitored by the absence of lesions as the ultimate read-out, thereby circumventing the necessity of an indirect challenge with luciferase-encoding pseudoviruses to detect neutralizing antibodies by *in vivo* imaging [30].

The preclinical animal model described here allows the investigation of virus-host interactions under defined *in vivo* conditions. Particularly, this study addresses important aspects which differentiate cutaneous from genital infections and might affect the efficacy of a vaccine against skin PV types. Firstly, will immunity conferred by VLP vaccination be effective against infections of the skin in a natural host? Secondly, what will be the outcome of vaccination when applied to animals with previously infected skin? In the case of a vaccine against cutaneous HPV types, this issue would be of the outmost importance because infections are acquired early in lifetime [24]. Thirdly, can a vaccine also protect against malignant skin tumors in its natural host? And finally, what is the influence of immunosuppression on the long-term efficacy of the vaccine? Answering these questions will be fundamental for the clinical development of a cutaneous HPV vaccine, given that the main targeted group of individuals would be OTR before undergoing systemic immunosuppression. In principle, the proof-of-concept of effective vaccination against a single skin PV type under the aforementioned conditions would represent the first step towards establishing a successful vaccination strategy in humans. In this regard, *Mastomys* provides an optimal model as the animals are latently infected with MnPV, exhibiting a similar tropism and pathogenicity as skin-associated HPV.

## Results

### Study design and immunization with MnPV VLPs

**Figure 1:**
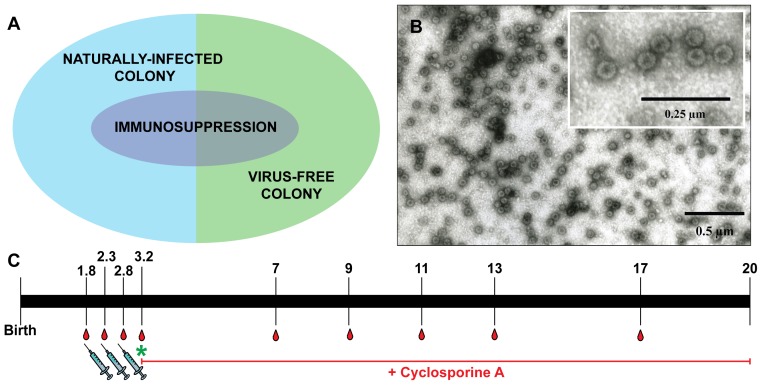
Overview of the vaccination study. **Panel A:** Vaccination was performed on both naturally infected *Mastomys* and on virus-free animals, which were subsequently infected. A subgroup of each colony also was kept under immunosuppressive conditions. **Panel B:** Electron micrograph showing MnPV VLPs with a size of 55 nm. **Panel C:** Time course of the vaccination study. Numbers indicate the time in months. Animals were vaccinated and bled as depicted. The green asterisk marks when the virus-free animals were experimentally infected. The red line indicates the duration of the treatment with cyclosporine A for the corresponding subgroups (for details, see Table 1).

To investigate the effect of MnPV VLP vaccination under different conditions, we used for immunization young animals derived from naturally infected *Mastomys* and also a virus-free colony that can be infected under defined experimental conditions. Based on the fact that MnPV is not transmitted *in utero* [28], virus-free *Mastomys* were obtained by hysterectomy [31]. Newborns were fostered by specific-pathogen-free (SPF) mice. The siblings were mated to establish a colony and their offspring analyzed at different ages for MnPV DNA in tissue biopsies, as well as for MnPV E2- and L1-specific seroconversion. Virus screening was performed by an established PCR protocol [23]. Samples were taken from potential infection sites [16], [28], namely the furred skin from the back, the eyelid, anogenital tissue and the tongue, which were all found to be negative. During the 3 years of existence of the virus-free colony, no MnPV-induced tumors were observed. After vaccination, subgroups from both cohorts were also immunosuppressed (Fig. 1A, see also Table 1 for details).

**Table 1:** Overview of the vaccination study.

Group	n	Treatment (age)				
		Vaccine (1.8 m)	Vaccine (2.3 m)	Vaccine (2.8 m)	Experimental infection (3.2 m)	Start of CsA feed (3.2 m)
A.I	52				+	
A.II	51	+	+	+	+	
A.III	18				+	+
A.IV	20	+	+	+	+	+
A.V	8	+	+	+		
B.I	33					
B.II	33	+	+	+		
B.III	20					+
B.IV	20	+	+	+		+

n = number of animals; m = month; CsA = Cyclosporine A.

A = groups from the virus-free colony (I = control; II = vaccinated; III = immunosuppressed control; IV = immunosuppressed vaccinated; V = non-infected vaccinated group).

B = groups of the naturally infected colony (I = control; II = vaccinated; III = immunosuppressed control; IV = immunosuppressed vaccinated).

Since our study involved a large cohort of animals which were to be followed up for more than one year, it was important to first define the optimal vaccination parameters (i.e, time, dosage, use of adjuvant, number of boosts). Groups of five 4-week-old *Mastomys* from the naturally infected colony were immunized subcutaneously with different vaccine formulations. The primary read-out for this pilot study was the generation of humoral immune responses against the MnPV major capsid protein. The induction of anti-L1 antibodies was measured by a newly set-up VLP-ELISA. All animals vaccinated with VLPs produced in baculovirus-infected insect cells (Fig. 1B) had a response above the calculated cut-off (Fig. S1). Since VLPs are highly immunogenic structures [32], the use of low doses of antigen was sufficient to elicit a response. Here, the increase from 5 to 25 µg VLP per dose (Fig. S1, groups 1 and 5) did not have a substantial impact on the anti-L1 response. Conversely, the effect of adjuvant addition in the formulation was significant, as the use of Sigma Adjuvant System (SAS) proved to be very effective (p<0.05 vs. no adjuvant; p<0.05 vs. aluminum hydroxide). Furthermore, two boosts after a first inoculation of VLPs+SAS proved to be optimal for inducing high antibody titers. Therefore, in all following experiments the vaccine was administered in a first dose of 10 µg VLPs formulated in PBS and SAS followed by two booster doses of 10 µg VLPs in PBS without adjuvant.

### Immunization with VLPs induces high titers of neutralizing antibodies

**Figure 2:**
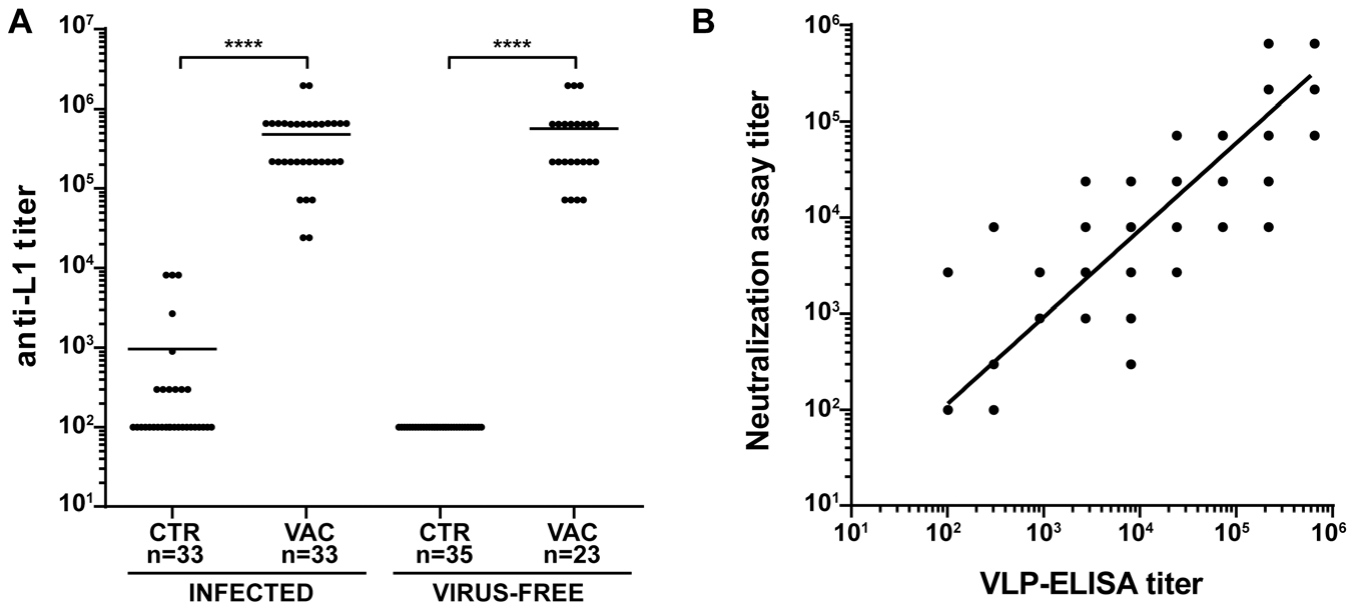
Humoral immune response to VLP vaccination. **Panel A:** Antibody titers against the major capsid protein of MnPV were measured by VLP-ELISA two weeks after the third vaccination. The end point titer was determined as the reciprocal of the highest serum dilution with an OD above the blanks. Sera were measured from animals of the naturally and experimentally infected colonies as indicated. Statistical significance was assessed by the Mann-Whitney test: ****, p<0.0001. CTR = unvaccinated controls; VAC = vaccinated animals. **Panel B:** Correlation between the titer of neutralizing antibodies and anti-L1 antibody titers measured by VLP-ELISA. Sera were obtained from animals of all groups (preimmune sera, n = 20; control animals, n = 32; vaccinated animals, n = 34; immunosuppressed control animals, n = 12; immunosuppressed vaccinated animals, n = 9). The correlation coefficient (r^2^) was 0.8919 and the slope of the regression line 0.9048, demonstrating a strong correlation between both methods. n: indicates the number of animals.

To investigate the effectiveness of the vaccine in our model, the study was divided in two main branches (Fig. 1A and Table 1): VLPs were administered to *Mastomys* from the naturally infected colony as well as to virus-free animals which were subsequently infected. Seroconversion of the naturally infected animals at the time of vaccination was assessed by the appearance of antibodies directed against the MnPV E2 protein (Fig. S2), known to be the earliest marker of infection [23]. Two weeks after completion of the vaccination schedule, the sera showed high anti-L1 titers by VLP-ELISA, whereby all animals responded to the vaccine both in the naturally infected and in the virus-free colony (geometric mean titer virus-bearing colony: 3.1×10^5^; geometric mean titer virus-free colony: 3.5×10^5^; range: 2.4×10^4^–1.9×10^6^) (Fig. 2A).

To test for the elicitation of neutralizing antibodies, we developed an *in vitro* neutralization assay which tests the ability of raised antibodies to prevent infection by MnPV pseudovirions that carry a reporter gene [33]. The system was first calibrated with two HPV L2-specific monoclonal antibodies (K4L2 and K18L2) which cross-neutralize several papillomavirus types [34]. Both antibodies displayed a high neutralization titer, showing proper sensitivity in our experimental setup (Fig. S3). In a next step, we monitored the ability of sera from animals of all groups to prevent infection in the MnPV pseudovirion assay. As depicted in Fig. 2B, the degree of neutralizing activity of the sera displayed a high correlation with the anti-L1 titers as assayed by VLP-ELISA (r^2^ = 0.8919, n = 113), regardless of the age of the animals as well as their infection and immune status (Fig. S4).

### Humoral immune responses to VLP vaccination are long-lasting and even maintained after chronic immunosuppression

**Figure 3:**
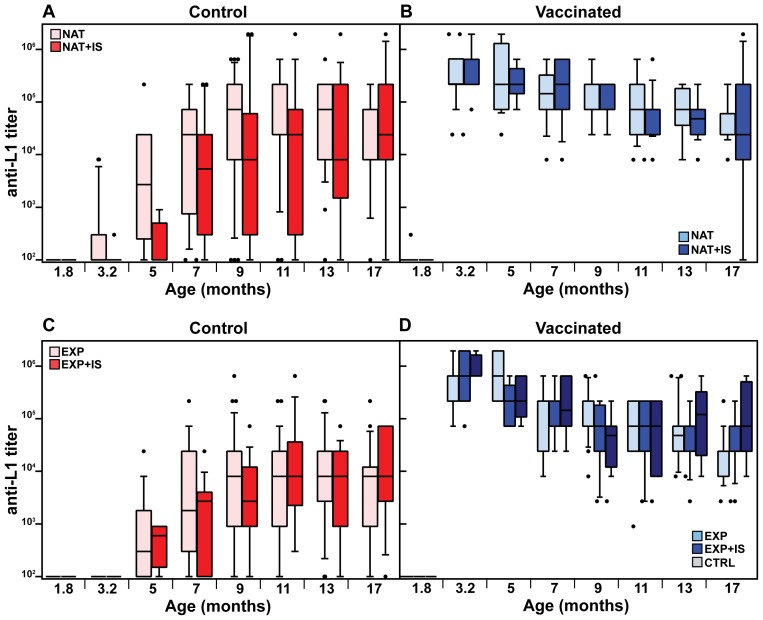
Time course of the anti-L1 antibody titer. The time course of antibody production was studied in the naturally infected colony, both in control (A) and vaccinated animals (B). Additionally, the same was measured in control (C) and vaccinated animals (D) from the former virus-free colony. Antibody titers were measured by VLP-ELISA at different time-points and are displayed as box plots. Boxes span the interquartile range and contain the median as a horizontal line. Outliers (•) are depicted outside the 10th and 90th percentile (whiskers). NAT = naturally infected colony; EXP = originally virus-free colony after experimental infection; IS = immunosuppressed animals; CTRL = vaccinated virus-free animals (for number of animals, see Table 1).

We next analyzed the time course of L1-induced antibodies in the naturally and experimentally infected colonies both under normal and immunosuppressed conditions. Substantial responses could be discerned at around 5 months in non-vaccinated controls, independently of whether the sera were derived from naturally or experimentally infected immunocompetent animals (Fig. 3A and C, pink bars). The natural immune response reached vaccination-like titers at an age of 5–7 months (Table S1). Considering the vaccinated cohorts (Fig. 3B and D, light blue bars), antibodies reached a geometric mean titer of 3.1×10^5^ and 3.5×10^5^ in both colonies after the third immunization, declining to 3.6×10^4^ and 1.9×10^4^ after 14 months, respectively. To exclude that the observed seroresponses in both groups were due to continuous boosting of the immune system caused by viral infection, thereby overlapping the profiles depicted in Fig. 3A and C, we also vaccinated a group of animals which remained virus-free during the whole study (Fig. 3D, grey bars). Anti-L1 antibody titers were stable over the same period, with a geometric mean titer of 8.5×10^5^ immediately after vaccination that dropped to 7.2×10^4^ after 14 months.

The specific antiviral humoral immune response was also assessed in animals undergoing systemic immunosuppression. To mimic this situation, cyclosporine A (CsA) was incorporated into food pellets as previously described [35], [36]. With this approach, immunosuppressive drugs can be given for an indefinite period of time without the animals suffering from the stress of daily intraperitoneal injections or gavage feeding. CsA concentration was measured in the heparinized blood both during the initial phase (574±132 ng/ml) and the maintenance phase (235±81 ng/ml) and was in accordance to the already reported immunosuppressive range for mice and humans [37], [38]. In order to assure that biologically active CsA concentrations were present, we additionally monitored *Mastomys*-specific interferon-γ (IFN-γ) expression in spleen cells of individual animals either under immunocompetent or immunosuppressed conditions by quantitative real-time PCR. IFN-γ expression was down-regulated by 70% in CsA-treated animals, indicating biologically active levels (Fig. S5).

Antibody titers after vaccination against L1 in the naturally infected or the virus-free colony, measured before immunosuppression (at 3.2 months of age, see Fig. 1C), reached similar levels (geometric means of 5.5×10^5^ and 6.2×10^5^, respectively) as in the immunocompetent groups at the same age (Fig. 3B and D, compare dark and light blue bars). Likewise, the drop of approximately one log in antibody titer (means of 1.6×10^4^ and 4.3×10^4^, respectively) after 14 months of immunosuppression showed that this treatment did not significantly affect the antibody response. Analyzing the course of L1-induced antibodies in the naturally and experimentally infected colonies after immunosuppression, antibody responses started at around 3–5 months and also increased in a time-dependent manner, as already revealed for immunocompetent animals (Fig. 3A and C, red bars).

### VLP vaccination effectively prevents the appearance of skin tumors

**Figure 4:**
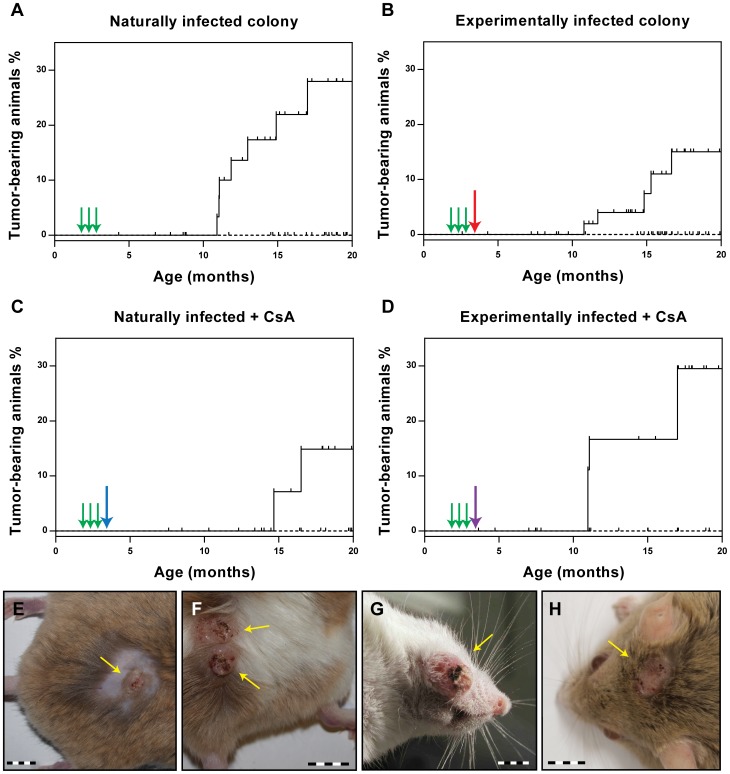
Incidence of virus-induced tumors. Immunocompetent and immunosuppressed animals were monitored over a period of 20 months for the occurrence of skin tumors (for number of animals, see Table 1). The Kaplan-Meier plots (**Panels A–D**) show the tumor incidence for each group in vaccinated (dashed line) and control animals (full line). Tumor-bearing animals displayed 1–4 skin tumors each (mean = 1.4). Green arrows indicate the time points of vaccination. The red arrow indicates the time of experimental infection, the blue arrow the start of the CsA feed, the purple arrow of both. The small vertical bars indicate censored animals which died for unknown reasons before tumor development. Differences between vaccinated and control animals were evaluated using the log rank test. The number of animals is given in Table 1. **Panels E/F:** Papillomas and keratoacanthomas in the lower back (infection site) of control animals from the experimentally infected colony. **Panels G/H:** Papillomas and keratoacanthomas arising in control animals from the naturally infected colony. Bars: 1 cm.

As the ultimate read-out for the vaccination efficacy, we evaluated the ability of the VLPs to prevent the appearance of skin tumors. Animals were examined monthly starting at the age of 7 months. After 13 months of observation (animal age: 20 months), none of the vaccinated animals had developed skin tumors in any of the vaccination groups. Conversely, the percentage of tumor-bearing animals at the end of the observation period was 28.0% for the naturally infected animals (p<0.0001 vs. vaccinated) and 17.5% for the experimentally infected colony (p<0.01 vs. vaccinated) (Fig. 4A and B). Thus, vaccination with VLPs can effectively prevent skin tumor formation, even when already infected animals are vaccinated. In the case of the virus-free *Mastomys*, which had been experimentally infected with a MnPV-containing wart extract in the lower back, the observed skin tumors appeared only in this area (Fig. 4E and F). In contrast, as described previously [23], lesions could be detected over the entire body in the naturally infected colony (Fig. 4G and H). Notably, tumors appeared at around 11 months in both groups of animals, independently of the mode of infection (Fig. 4A and B).

Also in immunosuppressed animals, a complete protection from tumor development was observed after VLP vaccination (Fig. 4C and D), whereas 29.5% of the experimentally infected *Mastomys* (p<0.05 vs. vaccinated) and 14.9% of the naturally-infected (ns vs. vaccinated; p = 0.0788) had tumors by the age of 20 months. This indicates that CsA treatment after vaccination does not affect the protection conferred by VLP vaccination, which is consistent with the presence of neutralizing antibodies even under immunosuppressive conditions (see Fig. 2B and Fig. 3).

### Histological examination of naturally and experimentally induced tumors

**Figure 5:**
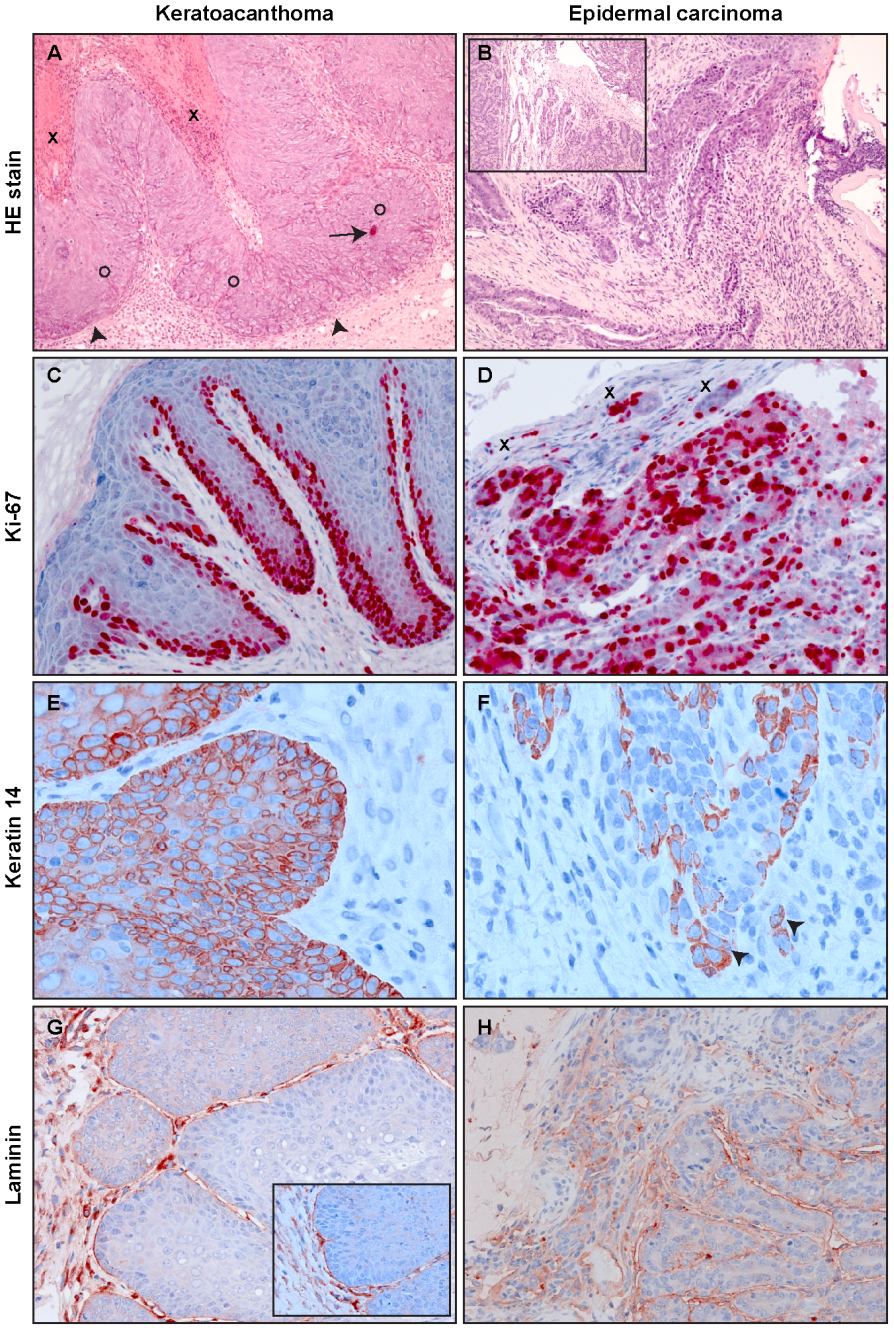
Tumor histology. A representative example of a virus-induced keratoacanthoma (A, C, E and G) and an epidermal carcinoma (B, D, F and H) is shown. Epidermal carcinomas appeared in one animal from the immunocompetent naturally infected colony and one animal from the immunocompetent experimentally infected colony. **Panel A:** Part of keratin filled craters (X) can be seen in the upper left. Irregular epidermal acanthotic proliferations (O) push into the dermis with preservation of a boundary (arrowheads). Apoptotic bodies can be seen (arrow). **Panel B:** Upper part of an epidermal carcinoma with ulceration, characterized by irregularly shaped strands of atypical keratinocytes invading the inflamed stroma. The inset shows deeper solid parts of tumor with high rates of mitosis and increased nuclear cytoplasmic ratios amongst other cellular atypical features. **Panel C:** At the lateral margin of a keratoacanthoma, a thickened highly proliferative basal layer can be seen by Ki-67 label. The upper layers also show some Ki-67 positivity. **Panel D:** In an epidermal carcinoma almost all cells are Ki-67-positive, showing the high proliferative rate of the tumor. Small tumor islets (X) invade the stroma. **Panel E:** Immunostaining against keratin 14 shows a succinct demarcation of proliferative epidermis from dermal stroma. **Panel F:** Keratin 14 immunohistology, showing the dropping off of tumor cells into the stroma (arrowheads). **Panel G:** By laminin immunohistochemistry, a separation of epidermal proliferation from the dermal stroma can be visualized. The inset shows a higher magnification. **Panel H:** Laminin staining is not continuous, constituting an additional criterion for the invasive nature of the epidermal carcinoma. Original magnification panels A, B and B-inset: 100×; panels C, D, G and H: 200×, panels E, F and G-inset: 400×.

In a next step, we examined the histological features of the skin tumors obtained in non-vaccinated animals after different modes of infection. As previously reported, most lesions in *Mastomys* are characterized as papillomas and keratoacanthomas, but also squamous cell carcinomas can be discerned [39], [40]. Indeed, the lesions found in our animals included not only benign, but also malignant tumors that can be classified as epidermal carcinomas. As depicted in Fig. 5A, keratoacanthomas show a thickened epidermis with principally maintained epidermal layers and a high proliferative index, as monitored by Ki-67 staining (Fig. 5C). In general the lesion does not infiltrate, as demonstrated by an intact basement membrane uniformly stained by antibodies directed against laminin (Fig. 5G). Also the keratin stain shows compact lobules of epidermal cells respecting the boundary to stroma (Fig. 5E). In contrast, an epidermal carcinoma obtained by experimental infection depicts, similarly to naturally induced malignant tumors, highly proliferating cells distributed over the whole tissue section (Fig. 5D). The basement membrane is no longer intact and keratin and Ki-67 positive singular cells can be visualized in the inflamed stroma (Fig. 5F and H). An increased nucleocytoplasmic ratio, a moderate nuclear pleomorphism and nuclear polycromasia can be seen, indicative of malignancy of the lesion. These data show that vaccination not only protects against benign but also from malignant skin tumors.

### VLP vaccination reduces the viral load in the skin

**Figure 6:**
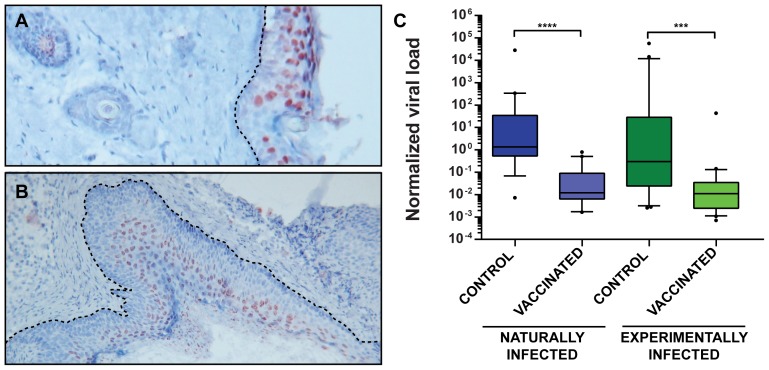
MnPV viral load in skin biopsies. **Panel A:** The red color shows viral genomes in the suprabasal layers of normal skin visualized by DNA *in situ* hybridization. The dotted line marks the basal membrane. **Panel B:** MnPV DNA *in situ* hybridization of a papilloma. Hyperproliferation of the epidermis can be observed, as well as basal and parabasal cells harboring MnPV DNA. Original magnification panel A: 80×; panel B: 40×. **Panel C:** qPCR analyses to detect viral DNA were performed by amplifying a fragment of the late L1 ORF. Data were normalized by relating the values to the copy number of β-globin and considering two copies of the gene as a cell equivalent. Tissue samples were taken from normal skin from animals from the naturally infected colony [control (n = 19), vaccinated (n = 19)] and the experimentally infected colony [control (n = 21), vaccinated (n = 22)]. Boxes span the interquartile range and contain the median as a horizontal line. Outliers (•) are depicted outside the 10th and 90th percentile (whiskers). Statistical significance was assessed by the Mann-Whitney test: ***, p<0.001; ****, p<0.0001. Median age for controls: 15.4 months (range: 8.8–20.4 months); vaccinated: 15.1 months (range: 6.7–21.3 months).

As reported previously, MnPV DNA can be found in the skin of virtually all animals older than 4 weeks in the naturally infected colony, suggesting an early transmission and long persistence [28]. Moreover, monitoring healthy skin or papillomas in older animals, the viral load increases in more differentiated regions (such as the *stratum spinosum* and *stratum granulosum*) to levels that could be detected by *in situ* hybridization (Fig. 6A and B). To test whether the viral load of the skin can be used as a further surrogate marker for vaccination efficacy, quantitative PCR of skin samples from tumor-free animals reaching the study end-point was performed. As presented in Fig. 6C, vaccinated animals had a lower viral load than unvaccinated controls, suggesting that vaccination with VLPs at a young age effectively prevents the increase in viral load, which in turn predicts which animals are prone to subsequent tumor formation [23].

### Protection is mediated by neutralizing antibodies

**Figure 7:**
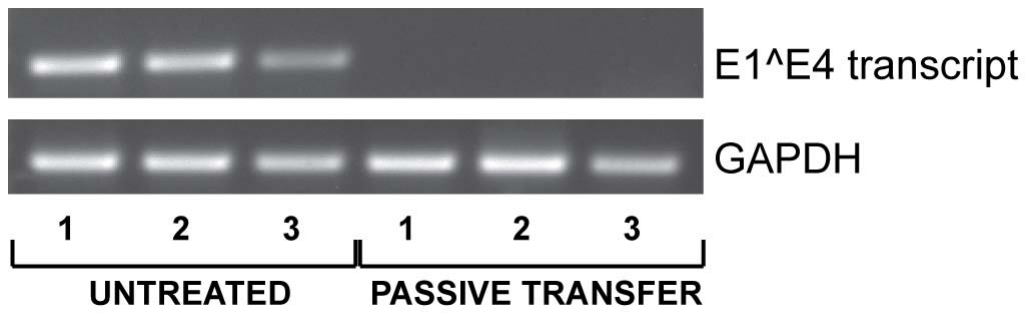
Inhibition of virus infection by passive immunization. To assess the role of neutralizing antibodies, polyclonal serum from vaccinated animals was injected intraperitoneally one day before challenge with MnPV infectious particles. After one week, RNA was extracted from the infected area to detect the MnPV specific E1∧E4 transcript by RT-PCR. GAPDH expression was used as internal control. Three animals were analyzed per group.

Since vaccinated animals displayed both a low MnPV load in the skin (Fig. 6C) and a complete protection from skin tumor formation (Fig. 4), we tested whether elicited antibodies were also protective under *in vivo* conditions. For this purpose, sera from vaccinated animals revealing a high antibody titer in the *in vitro* neutralization assay were pooled and administered intraperitoneally to virus-free *Mastomys* 24 hours prior to experimental infection. One week after passive immunization, RNA was extracted from infected skin areas and analyzed by RT-PCR for the appearance of the prevalent E1∧E4 transcript found in a recently performed *Mastomys* transcriptome analysis from a keratoacanthoma (Vinzón *et al.*, manuscript in preparation). As shown in Fig. 7, transfer of anti-VLP immune sera apparently prevented the virus from infecting the skin, as evidenced by the lack of the corresponding MnPV transcript at the site of infection. This result indicates that immune serum obtained from VLP vaccinated animals can protect from experimental infection by transmission of neutralizing antibodies.

## Discussion

The currently available HPV vaccines are a major breakthrough in the fight against HPV-associated genital cancers and proved the potential of prophylactic vaccines to prevent HPV-caused disease. These vaccines were developed on the basis of preclinical data obtained from animal models of PV infection [41]. Although valuable, these systems (namely the bovine papillomavirus, the canine oral papillomavirus or the cottontail rabbit papillomavirus models) present inherent difficulties that make their use in the laboratory technically challenging. Hence a small rodent model of papillomavirus infection would be beneficial for the testing of prophylactic vaccines as well as for the development of therapeutic approaches. In this regard, the multimammate rodent *Mastomys coucha* represents a unique outbred model, being naturally infected with both cutaneous and mucosal PV types which are etiologically linked to the formation of tumors [16]. We report here that a VLP-based vaccine against a cutaneous papillomavirus can effectively prevent the appearance of naturally and experimentally induced skin tumors, both under immunocompetent and immunosuppressive conditions, thus providing the basis for the implementation of vaccination strategies against cutaneous HPVs in OTR.

For the vaccine formulation, VLPs were chosen on the basis of their previous success in eliciting a protective immune response against other PVs in animal models and in humans. VLPs have the advantage of generally inducing high titers of neutralizing antibodies, which represent the major effector mechanism of most preventive viral vaccines, particularly in those against genital high-risk HPV types [42]. We could show a strong correlation between the total IgG levels against the L1 capsid protein as measured by VLP-ELISA and the amount of antibodies which are able to effectively neutralize the virus (Fig. 2B). Therefore, titers assessed by VLP-ELISA are reliable markers of a protective immune response.

Using both serological methods [23] and direct detection of viral DNA [28], previous studies have shown that natural infection with MnPV can occur earlier than four weeks after birth. This could hamper the evaluation of vaccination efficacy in preventing primary infection, since the actual time point of infection cannot be predicted for single cases. Therefore, a virus-free *Mastomys* population was established in order to assess the effect of vaccination on *naïve* animals. On the other hand, a particular advantage of the multimammate mouse is that the vaccine can be studied in a naturally infected colony, thus allowing the monitoring of immunization success in already virus-positive animals. This is of particular importance in the context of a human infection, in which some cutaneous HPVs are acquired early in childhood [24]. The currently licensed vaccines targeting HPV have not shown any evidence of accelerated clearance in patients who were DNA-positive at baseline for the type considered in the evaluation [13]. In our preclinical study, tumor formation was completely prevented even in previously infected animals, although VLP vaccination was not sufficient to completely clear PV infection in the skin (Fig. 6C). Hence, administration of VLPs may have a “therapeutic” effect by preventing virus spread during early infection and therefore avert tumor development.

Cutaneous immune surveillance is typically initiated by professional antigen-presenting cells which encounter the antigen in the skin and activate *naïve* T cells that finally orchestrate a proper adaptive immune response [43]. Although natural and experimentally infected control animals also raise a humoral response (Fig. 3A and C) resulting in the induction of neutralizing antibodies (Fig. 2B), the appearance of tumors cannot be prevented (Fig. 4A and B). Here, the immune system is apparently not able to handle early bursts of replication and high viral load accumulation that finally lead to a skin lesion. In fact, comparing the viral load in normal skin of vaccinated and control animals (Fig. 6C) revealed that the latter generally harbor more copies of the virus. This can also be visualized by *in situ* DNA hybridization where the amount of MnPV focally increases in cells of the *strata spinosum* and *granulosum* (Fig. 6A). Conversely, considering a papilloma, a more even distribution of cells with highly amplified MnPV DNA could be discerned (Fig. 6B). Consistent with earlier studies, viral persistence and high viral load correlated with the development of skin papillomas and keratoacanthomas [28]. Hence, high copy number may be a general feature of productive infections [44]. On the other hand, high viral load is also accompanied by enhanced oncogene expression that could finally predispose cells towards malignancy [45].

The novel finding that vaccination minimizes the viral load by at least 10–20-fold (Fig. 6C) clearly suggests that prevention of tumor development is due to the diminished efficiency of viral spread after an early infection. As it was already proposed for genital types, this effect is most likely conferred by neutralizing antibodies that in turn block *de novo* initiated productive viral cycles by preventing reinfection of cells in traumatized epithelium [46]. In fact, as demonstrated by passive transfer of sera from vaccinated to *naïve* animals, infection of the skin can be prevented (Fig. 7), showing that neutralizing antibodies directed against MnPV are sufficient for protection.

The way by which neutralizing antibodies can reach the basal epidermal layer in order to control infection is still unclear. For IgG antibodies reaching the genital mucosa, two transport mechanisms have been described: transudation mediated by the neonatal Fc receptor [47] and exudation of systemic antibodies at the site of infection. In the case of the skin, transudation seems unlikely and an exudation mechanism would be favored. This is in agreement with the fact that, to enter basal cells, papillomaviruses first have to bind to the basement epithelial membrane at sites where the epithelium is traumatized [28], [48]. Notably, it has been proposed that exudation might be responsible for the observed protection in the genital model, as clinical trials reported excellent protection from genital warts, which occur on cornified skin where protection by transuded antibodies is also unlikely to occur [12]. On the other hand, although it was previously thought that B cells and IgGs cannot enter the skin, there is accumulating evidence that exceptions exist, especially during chronic inflammatory processes or B cell malignancies. Firstly, although poorly understood, the skin can be infiltrated by a subset of B cells that connect innate with adaptive immunity [49]. Secondly, high-resolution two-dimensional immunoblotting assays revealed the presence of IgGs in the upper lesional epidermis of psoriasis plaques [50], also known to be positive for cutaneous HPV types [51]. It remains to be determined how the exact spatial-temporal mechanism(s) that finally protects against a papillomavirus infection in the skin operates.

Another important issue to address during the development of a vaccine against cutaneous PVs is whether protection can be still conferred to immunosuppressed patients. This matter is of paramount importance, when considering such a vaccine for OTR who undergo chronic systemic immunosuppression. Vaccination before transplantation is recommended for many other preventable diseases and has been reported to be effective, with a lower rate of success when patients are immunized after immunosuppression [52], [53]. Chronic immunosuppression with CsA did not significantly affect the antibody response (Fig. 3), as it was previously reported for CsA-treated rabbits during rabbit oral papillomavirus infection [54] and in vaccination studies involving transplant patients [55]. In accordance to this and supporting a role for neutralizing antibodies in the protection from tumor formation, no lesions appeared in the vaccinated animals which underwent immunosuppression. Conversely, animals which were not vaccinated developed tumors (Fig. 4), as was seen in the immunocompetent animals. Although tumor incidence in immunosuppressed *Mastomys* was higher in experimentally infected and lower in naturally infected animals, neither of these differences were significant. The size of the immunosuppressed groups in this study was not designed to assess whether there is an effect of immunosuppression on tumor incidence and, therefore, we cannot draw any conclusions in this regard. Additionally, the administration regimen of CsA could have played a role in the lack of increased tumor formation, as it was recently shown in a UV-irradiated mouse model of skin cancer that CsA administered in a bolus rather than continuously is able to increase the number of tumors [56]. Nonetheless, we show that this vaccination strategy has credible potential in the difficult fight against skin lesions in immunosuppressed patients, especially those occurring in organ transplant recipients.

Given the variety of cutaneous HPV types that could be involved in the development of non-melanoma skin cancer in humans (such as HPV23, 38 and the EV types HPV5 and HPV8) [57], a broadly protective HPV vaccine would be ideal. Anti-L1 neutralizing antibodies are known to be highly HPV-type specific [12]. The papillomavirus minor capsid protein L2, however, contains a major cross-neutralizing epitope that could be used to develop a second generation vaccine [30]. We are currently investigating whether administration of an L2-vaccine is as effective in preventing skin tumors in *Mastomys coucha* as its L1 counterpart.

In summary, our data provide the first evidence that a vaccine targeting a cutaneous papillomavirus can effectively prevent the appearance of skin tumors even in animals which are already infected or will undergo systemic immunosuppression. Our results provide the basis for the clinical development of potent vaccination strategies against cutaneous HPV infections.

## Materials and Methods

### Animals

*Mastomys coucha* from the DKFZ breeding colony were maintained under conventional conditions (21–24°C, 55% relative humidity with 12–16 air changes per hour, mouse breeding diet and water *ad libitum*). Animals were checked monthly for the appearance of tumors. When tumors reached a size of 2 cm, the animals were sacrificed and blood was taken by cardiac puncture. Every two months, blood was taken from the submandibular vein. Animals were monitored throughout their whole lifetime until they had to be sacrificed for reasons of tumor development or decrepitude. Two weeks after the last vaccination dose (age: 3.2 months), animals in the immunosuppressed groups were switched to the same mouse chow supplemented with cyclosporine A (CsA, pharmaceutical grade powder; Fagron, Barsbuettel, Germany) at a concentration of 250 mg/kg during the first 3 months of treatment and 125 mg/kg for the rest of the animal's life. Higher doses were used in the beginning to reflect the level of immunosuppression administered immediately after organ transplantation in OTR, which is typically reduced after a few months [58]. The drug–mouse chow premix was cold pelleted according to standard procedures by SNIFF Spezialdiaeten (Soest, Germany). CsA concentration was measured in heparinized blood by HPLC (University Hospital Regensburg Clinical Laboratories) to confirm immunosuppressive levels were reached.

### Ethics statement

*M. coucha* at the DKFZ are maintained in compliance with German and European statutes and all animal experiments were undertaken with the approval of the responsible Animal Ethics Committee (Regional Council of Karlsruhe, Germany; 35-9185.81/G-124/08).

### Generation of a virus-free colony

Hysterectomies were performed on pregnant *Mastomys* under sterile conditions [31]. The offspring (four females and one male) were nursed by foster specified pathogen-free (SPF) mice (*Mus musculus*), kept in a specific pathogen free isolator unit at the DKFZ. From these animals, a virus-free colony was established. Testing for anti-MnPV and McPV2 antibodies as well as for viral DNA was regularly done on the progeny as described [23].

### Experimental infection

A wart extract containing infectious MnPV particles was obtained by grinding a frozen papilloma in a Mikro-Dismembrator S (Sartorius) and resuspending the homogenate in PBS. Before experimental infection, *Mastomys* from the virus-free colony were anaesthetized with 2.5% isoflurane. A 1 cm diameter patch on the back was shaved and gently scarified using a scalpel blade and 20 µL of the wart extract was applied to the patch. Animals were placed on their abdomens until the virus suspension dried before returning them to their cages.

### DNA isolation from dissected tissues

Animals were sacrificed by cervical dislocation and dissected tissue samples were frozen at −20°C. To avoid cross-contamination among different tissues, surgical instruments were changed after every sample dissection. The DNA was extracted as previously described [23].

### Quantification of the viral load and DNA *in situ* hybridization (ISH)

Quantification of MnPV DNA was performed with the iTaq Universal SYBR Green Supermix (BioRad) using 50 ng of total DNA per reaction, following the manufacturer instructions. Detection was done with the CFX96 real time PCR detection system (Bio-Rad). Binding sites of MnPV primers were located within the L1 genes (forward primer: 5′-ACGGCAACTCATGCTTCTTC-3′, reverse primer: 5′-CTCTGTGCCTGTCCATCCTT-3′). To determine the number of input cell equivalents, the single-copy-number gene β-globin was validated and quantified (forward primer: 5′-ACCATGGTGCACCTTACTGAC-3′, reverse primer: 5′-TCCAGGCACCCAACTTCTAC-3′). MnPV DNA copy numbers were determined in duplicate by using standard curves generated in the same PCR run with a standard containing MnPV and β-globin plasmids. Sensitivity was 5 copies of MnPV DNA per sample and quantification was linear from 5 to 5×10^8^ copies MnPV. MnPV DNA load was defined as the number of MnPV DNA copies/2 β-globin copies [59]. ISH was performed as previously described [28].

### Histological and immunohistochemical analysis

Tissue sections were fixed in 4% buffered formaldehyde, embedded in paraffin, cut at 3 µm and stained by hematoxylin eosin or used for immunostaining. Immunohistochemistry was performed for keratin 14 (K14), laminin and the proliferation marker Ki-67. K14 was labeled with a guinea pig antibody (k14.2; Progen, Heidelberg, Germany) at a dilution of 1∶100 and laminin was labeled with a 1∶50 dilution of rabbit polyclonal antibody donated by Dr. Stark, DKFZ, Heidelberg (ln(537)); Ki-67 was detected with a mouse monoclonal IgG1 (NCL-L-Ki67-MM1; Novocastra) used at a dilution of 1∶100. Sections stained with k14.2 were pretreated by cooking for 20 minutes in citrate buffer, pH 6.0 and sections stained for Ki-67 were additionally pretreated with proteinase K for 15 minutes at 37°C. Staining was performed by streptavidin coupled to horseradish peroxidase or alkaline phosphatase, as described [60].

### VLP production

Sf9 cells (Invitrogen) were kept as described elsewhere [61]. Full-length MnPV L1 gene was cloned into the pVL1393 vector (Invitrogen) by PCR amplification. The construct was confirmed by DNA sequencing. For the production of recombinant MultiBac AcNPVs, 2 µg of the pVL1393 derived recombinant L1-encoding plasmid and 1 µg of MultiBac bacmid DNA were co-transfected by calcium phosphate precipitation using 1 ml Sf9 transfection buffer. After incubation for 5 h at 27°C and 5% CO_2_, the cells were washed twice and maintained in Grace's medium under the same conditions for one week. The recombinant Ac virus was amplified before its employment for a productive infection of TN High Five cells and the titer determined by a plaque assay.

For VLP production, 2×10^8^ TN High Five cells were infected with wild-type or recombinant baculovirus at a MOI of 2. Three days post-infection, cells were harvested and lysed by sonication. Subsequently, the lysate was cleared by centrifugation, layered onto a two-step gradient with 14 ml of 40% sucrose on top of 8 ml of a 57.5% CsCl solution and centrifuged for 3 h at 96,500× g at 10°C using a SW32 rotor (Beckman). The interphase was collected and transferred into a Quick-seal tube (Beckman). A CsCl gradient was produced by a 16 hour-centrifugation at 184,000× g at 20°C in a Sorval TFT 65.13 rotor and fractionated into 1 ml aliquots. Purity and L1 content of the collected fractions were assessed by SDS-PAGE and Coomassie staining. The peak fractions were pooled, dialyzed against 50 mM Hepes (pH 7.4, 0.3 M NaCl), and cleared from residual debris by centrifugation at 20,000× g for 10 min at 4°C. L1 protein concentrations were determined using image densitometry software ImageJ (http://rsb.info.nih.gov/ij/) and bovine serum albumin as standard for the Coomassie-stained SDS-PAGE gel. The capsid quality was verified by electron microscopy. We produced a large batch of high quality VLPs sufficient for the whole vaccination study. Fractions were analyzed by electron microscopy four months and one year after storage, which revealed a robust stability of the particles.

### Electron microscopy

Electron microscopy (EM) was performed by Birgit Hub (DKFZ, Heidelberg) using a Zeiss EM-10 transmission electron microscope. The structure and quality of particles derived from VLP preparations were analyzed by negative staining. VLPs (approximately 100 ng) were applied on carbon coated grids, stained with 2% uranyl acetate and analyzed in the EM at 20,100 fold magnification.

### VLP immunization

Animals were immunized at an age of 8 weeks with 10 µg dialyzed VLPs in the presence of the Sigma Adjuvant System (SAS), containing monophosphoryl lipid A (MPL) and synthetic trehalose dicorynomycolate in squalene and Tween80. The formulations were prepared as suggested by the manufacturer. The vaccine was delivered in VLP vaccine buffer (50 mM Hepes, pH 7.4, 0.3 M NaCl) and a volume of 200 µl was injected subcutaneously in a skin fold of the neck. Control animals were immunized with 200 µl of vaccine buffer alone.

### MnPV VLP-ELISA

For MnPV VLP-ELISA, 96-well plates (Polysorp, Nunc) were coated overnight at 4°C with 1 µg/ml VLPs in 50 mM carbonate buffer pH 9.6 and blocked the next day with casein blocking buffer (0.2% casein in PBS, 0.05% Tween 20). Plates were subsequently incubated 1 h at room temperature with three-fold dilutions of the sera in casein blocking buffer. Plates were washed and bound specific *Mastomys* IgG was detected with a HRP-conjugated goat anti-mouse IgG antibody (heavy+light chain; Promega, Mannheim, Germany) diluted 1∶10,000 in blocking buffer. Color development was performed by addition of 0.1 mg/ml tetramethylbenzidine (Sigma, Steinheim, Germany) and 0.006% H_2_O_2_ in 0.1 M sodium acetate pH 6 (100 µl/well). After 8 min, the enzyme reaction was stopped with 50 µl of 1 M sulfuric acid per well and the absorbance was measured in an automated microplate reader (Labsystems Multiskan; Thermo Fisher Scientific, Waltham, USA) at a wavelength of 450 nm. Antibody titer represents the last reciprocal serum dilution above blank.

### Pseudovirion preparation

Pseudovirions were generated as previously described [62] with some modifications. 293TT cells were cotransfected with a plasmid encoding humanized MnPVL1 and L2 genes and a Gaussia luciferase reporter gene plasmid using TurboFect transfection reagent (Fermentas) according to the manufacturer's instructions. Cells were incubated at 37°C, 5% CO_2_ for 72 h, harvested and resuspended in the same volume of DBPS supplemented with 0.5% of Brij 58 (Sigma) and 1% RNAse A/T (Fermentas). Cells were incubated overnight at 37°C under rotation to allow pseudovirion maturation. On the next day, pseudovirus were extracted in 0.8 M NaCl and incubated with 250 U of benzonase (Merck) for 1 h at 37°C. The pseudovirions were subsequently purified by an Optiprep (Sigma) gradient. Fractions with highest reporter gene expression were pooled and aliquots were stored in siliconized tubes at −80°C.

### *In vitro* pseudovirion-based neutralization assay

The neutralization assays were performed as previously described [62] with some modifications. Briefly, 60 µl of diluted polyclonal or monoclonal antibodies were combined with 40 µl of diluted pseudovirus stocks and incubated at room temperature for 20 min. Next, 50 µl HeLaT cells (HeLaT-clone 4) [63] (2.5×10^5^ cells/ml) were added to the pseudovirus-antibody mixture and incubated for 48 h at 37°C, 5% CO_2_. The amount of secreted Gaussia luciferase in 10 µl of cell culture medium was determined using coelenterazine substrate and Gaussia glow juice (PJK, Germany) according to the manufacturer's instructions. A microplate luminometer (Synergy 2, BioTek) was used to measure the samples 15 min after substrate addition.

### *In vivo* virion-based neutralization assay

To assess the role of neutralizing antibodies on the skin infections, we performed a virion neutralization assay. Basically, a patch on the back of each anesthetized *Mastomys* was shaved with an electric razor and slightly scarified with a scalpel blade. Polyclonal serum from 5 vaccinated animals was pooled and diluted 1∶10 in PBS and 200 µL injected intraperitoneally one day before challenge with 20 µL of a wart extract containing MnPV infectious particles. One week after challenge, the animals were sacrificed and samples were taken from the treated areas of the skin.

Total cellular RNA was extracted by using the RNeasy Kit (QIAGEN), according to the manufacturer's instructions. To eliminate all traces of viral DNA to avoid false positive signals by RT-PCR, the RNA was additionally treated with DNase I (QIAGEN). Reverse transcription was performed with the reverse transcriptase SuperScript II (Invitrogen), according to the manual. To evaluate the infection with MnPV virions, the abundant E1∧E4 transcript was detected (Vinzón *et al.*, unpublished) after 32 PCR cycles with a forward primer spanning the splicing junction at nucleotides 808∧3144 and an appropriate reverse primer (forward primer: 5′-TGAAGAAGCTCTACACCGCA-3′, reverse primer: 5′-GTCTCCTCCTTTCGGGTGC-3′). As a control, *Mastomys* GAPDH transcript was determined after 25 PCR cycles (forward primer: 5′-CTTCATTGACCTCAACTACATGGTC-3′, reverse primer: 5′- CACAGTCCATGCCATCACTGC-3′).

### Statistical analysis

Prism 6.0 (GraphPad Software) was used for data analysis and graphic representation. Statistical analysis for comparisons of antibody titer of viral load was performed with the non-parametric Mann-Whitney U test. The distribution functions describing tumor incidence were determined using the Kaplan-Meier estimator. Differences in tumor incidence times between the different groups were evaluated using the log rank test.

## Supporting Information

Figure S1 **The VLP vaccine pilot study.** *Mastomys* from the virus-bearing colony (n = 5/group) were immunized subcutaneously with different vaccine formulations. For this purpose, we used 4-week-old animals to rule out false positive results in terms of L1 seroresponses arising from the natural infection (1). Conversely, to eliminate false negative results, younger animals were excluded due to the fact that their immune system might not be fully developed. The first VLP injection was applied subcutaneously, followed by booster immunizations 2 and 4 weeks later. Antibody titers against L1 were measured by VLP-ELISA 2 weeks after each vaccination dose. Alu = Aluminium hydroxide; SAS = Sigma Adjuvant System, containing monophosphoryl lipid A and synthetic trehalose dicorynomycolate. Open, grey and black circles: two weeks after the first, second and third immunization, respectively.
(TIF)

Figure S2 **E2 serology in 8-week-old animals.** Sera were collected from naturally infected (n = 119) and virus-free (n = 105) animals at an age of 8 weeks, prior to the initiation of the vaccination protocol. Antibody titers against E2 antigen were measured by a previously established GST-capture ELISA (1). Boxes comprise the titers falling in the range from the 25th to the 75th percentile, the line within shows the median. Outliers (•) are depicted outside the 5th and 95th percentile (whiskers). Anti-E2 antibodies revealed that most of the animals of the naturally infected colony were already infected at the time when vaccination started.
(TIF)

Figure S3 **Neutralization activity of cross-protecting antibodies.** K4L2 and K18L2 are monoclonal antibodies directed against the HPV16 L2 peptide 20–38 (2). Due to their broad cross reactivity, K4L2 and K18L2 were used to validate the *in vitro* neutralization assay against MnPV pseudoviruses (see *Material and Methods*). Whiskers represent the SEM (n = 2).
(TIF)

Figure S4 **Correlation between the titer of neutralizing antibodies (y-axis) and anti-L1 antibody titers measured by VLP-ELISA (x-axis).** **Panel A:** Naturally infected control animals (n = 19). **Panel B:** Naturally infected vaccinated animals (n = 20). **Panel C:** Experimentally infected control animals (n = 13). **Panel D:** Experimentally infected vaccinated animals (n = 14). **Panel E:** Naturally infected immunosuppressed control animals (n = 6). **Panel F:** Naturally infected immunosuppressed vaccinated animals (n = 9). **Panel G:** Experimentally infected immunosuppressed control animals (n = 6). **Panel H:** Experimentally infected immunosuppressed vaccinated animals (n = 6). The red line depicts the linear correlation between the neutralizing and the anti-L1 titer for the 113 sera (Fig. 2B). n: indicates the number of animals.
(TIF)

Figure S5 **Effect of CsA feeding on the expression of IFN-γ.** Spleens were dissected from *Mastomys* following a normal diet (•) or receiving food containing a low concentration (125 mg/kg) of CsA (▪). Splenocytes were isolated as described elsewhere (3) and stimulated with 2 µg/mL Concanavalin A (Sigma) for four days. RNA was extracted by using the RNeasy Kit (QIAGEN) according to the manufacturer's instructions. To eliminate all traces of viral DNA in order to avoid false positive signals by RT-PCR, the RNA was additionally treated with DNase I (QIAGEN). Reverse transcription was performed with the reverse transcriptase SuperScript II (Invitrogen) according to the manual. Quantification of IFN-γ transcripts was performed with the iTaq Universal SYBR Green Supermix (Bio-Rad), following the manufacturer instructions. Detection was done with the CFX96 real time PCR detection system (Bio-Rad). Primers were specifically designed to bind *Mastomys* IFN-γ transcripts (forward primer: 5′-CTGTTACTGCCAAGGCACAC-3′, reverse primer: 5′-CATCCTTTTGCCAGTTCCTC-3′). Data was normalized by the amount of β-actin transcripts in the same sample (forward primer: 5′-GAAGAGCTATGAGCTGCCTGAC-3′, reverse primer: 5′-GTTTCATGGATGCCACAGGA-3′).
(TIF)

References S1 **Supporting information references.**
(DOCX)

Table S1 **Statistical analysis of anti-L1 antibody titers.** A non-parametric Kruskal–Wallis analysis followed by a Dunn's post hoc multiple comparison test was performed to compare all groups in the study. Graphpad Prism was used for the analysis.
(DOCX)
