# Supplementary figures and images for: Protective Vaccination against Papillomavirus-Induced Skin Tumors under Immunocompetent and Immunosuppressive Conditions: A Preclinical Study Using a Natural Outbred Animal Model
Source: PLoS Pathog. 2014 Feb 20;10(2):e1003924. doi: 10.1371/journal.ppat.1003924 (PMC3930562; doi:10.1371/journal.ppat.1003924)

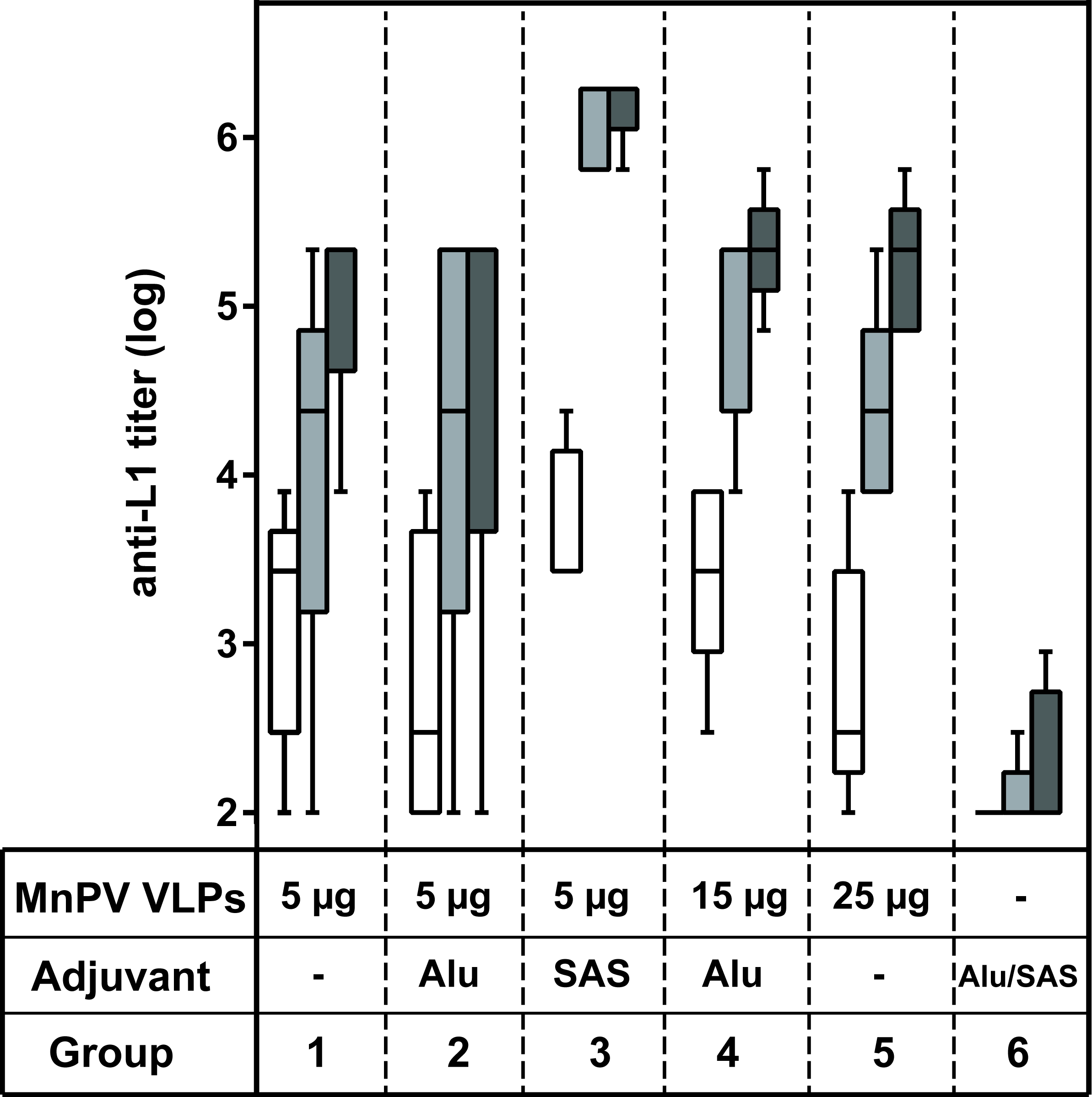

Supplement: Figure S1 — The VLP vaccine pilot study. Mastomys from the virus-bearing colony (n = 5/group) were immunized subcutaneously with different vaccine formulations. For this purpose, we used 4-week-old animals to rule out false positive results in terms of L1 seroresponses arising from the natural infection (1). Conversely, to eliminate false negative results, younger animals were excluded due to the fact that their immune system might not be fully developed. The first VLP injection was applied subcutaneously, followed by booster immunizations 2 and 4 weeks later. Antibody titers against L1 were measured by VLP-ELISA 2 weeks after each vaccination dose. Alu = Aluminium hydroxide; SAS = Sigma Adjuvant System, containing monophosphoryl lipid A and synthetic trehalose dicorynomycolate. Open, grey and black circles: two weeks after the first, second and third immunization, respectively. (TIF) [file ppat.1003924.s001.tif]

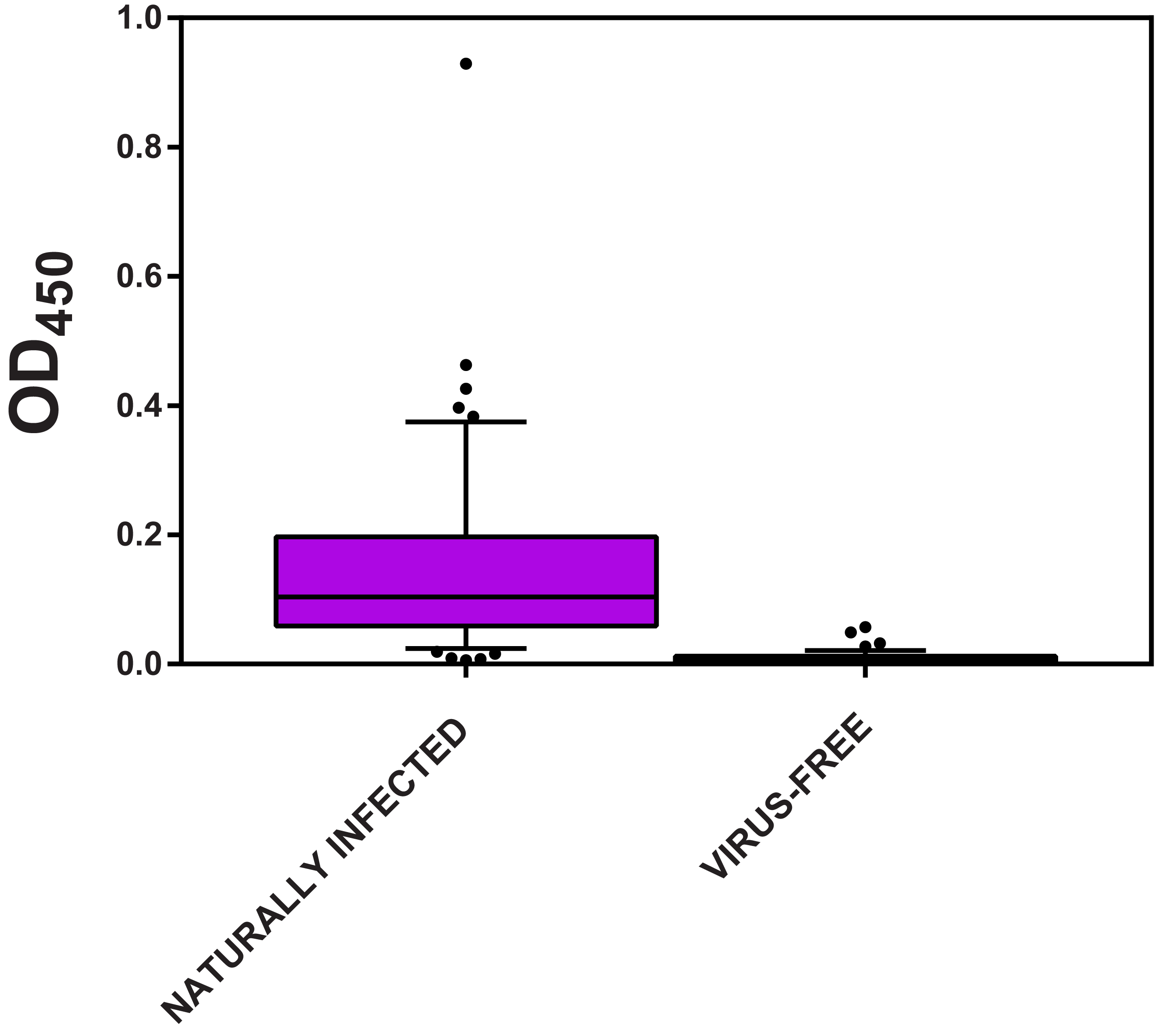

Supplement: Figure S2 — E2 serology in 8-week-old animals. Sera were collected from naturally infected (n = 119) and virus-free (n = 105) animals at an age of 8 weeks, prior to the initiation of the vaccination protocol. Antibody titers against E2 antigen were measured by a previously established GST-capture ELISA (1). Boxes comprise the titers falling in the range from the 25th to the 75th percentile, the line within shows the median. Outliers (•) are depicted outside the 5th and 95th percentile (whiskers). Anti-E2 antibodies revealed that most of the animals of the naturally infected colony were already infected at the time when vaccination started. (TIF) [file ppat.1003924.s002.tif]

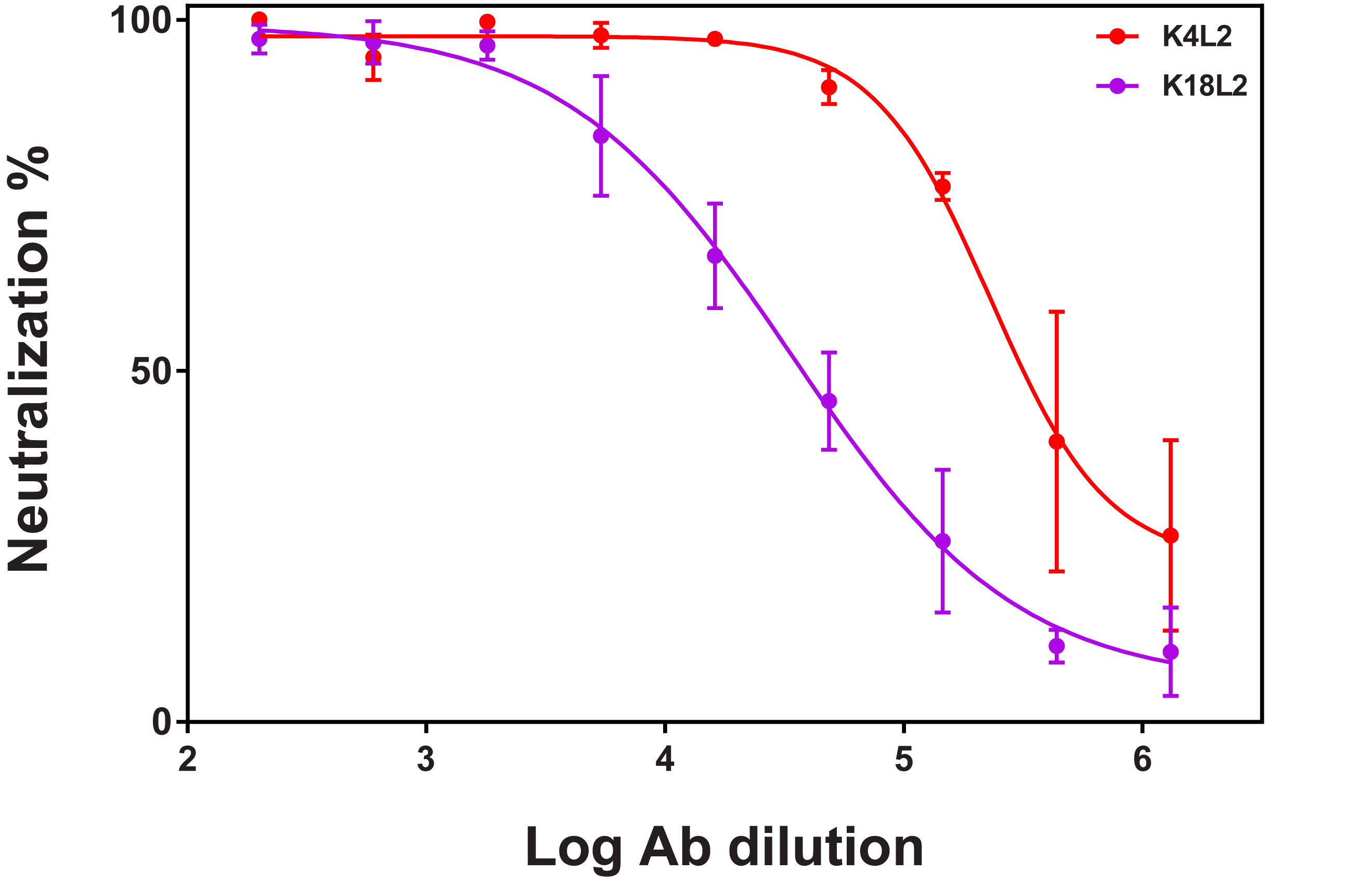

Supplement: Figure S3 — Neutralization activity of cross-protecting antibodies. K4L2 and K18L2 are monoclonal antibodies directed against the HPV16 L2 peptide 20–38 (2). Due to their broad cross reactivity, K4L2 and K18L2 were used to validate the in vitro neutralization assay against MnPV pseudoviruses (see Material and Methods). Whiskers represent the SEM (n = 2). (TIF) [file ppat.1003924.s003.tif]

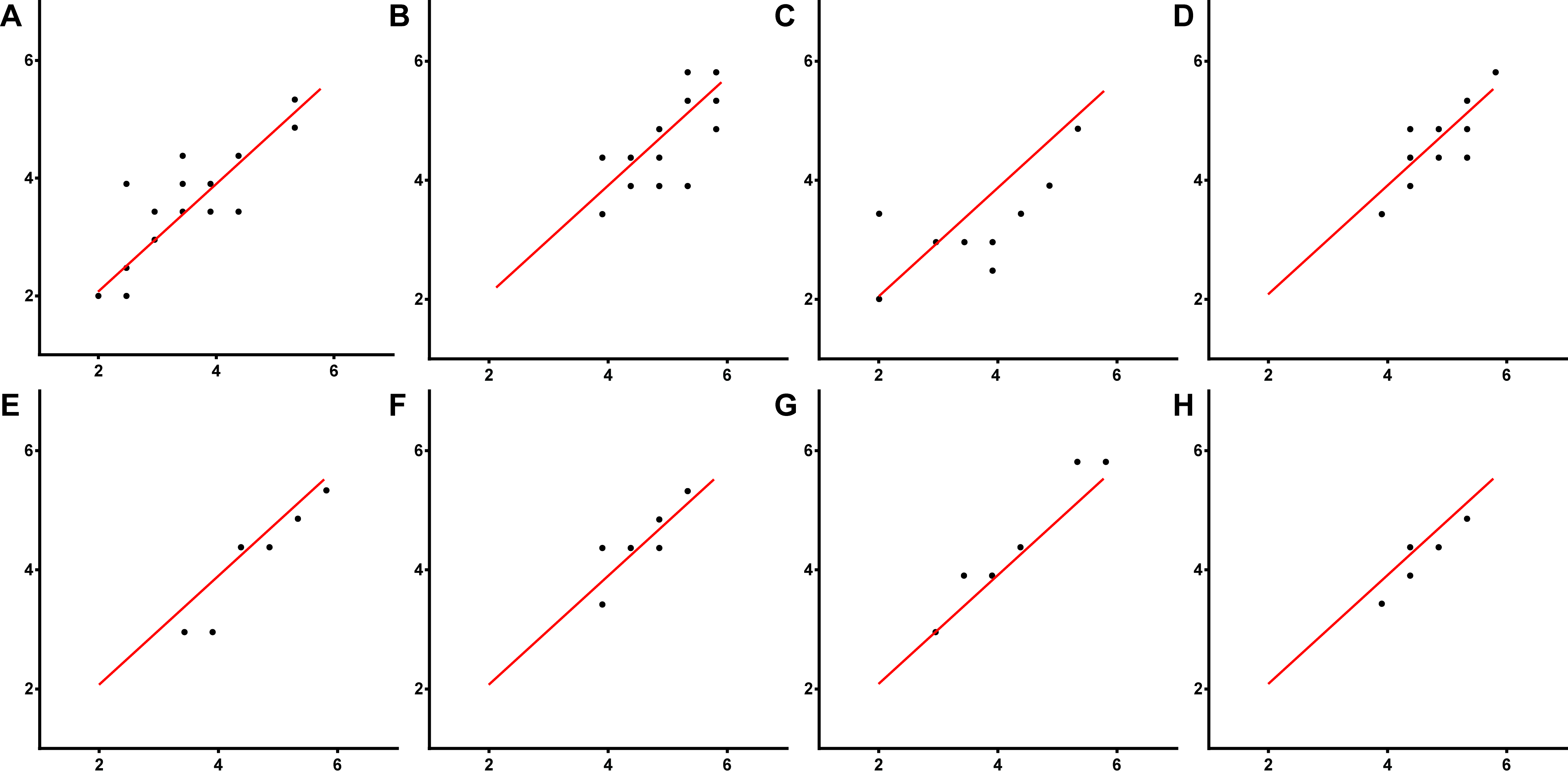

Supplement: Figure S4 — Correlation between the titer of neutralizing antibodies (y-axis) and anti-L1 antibody titers measured by VLP-ELISA (x-axis). Panel A: Naturally infected control animals (n = 19). Panel B: Naturally infected vaccinated animals (n = 20). Panel C: Experimentally infected control animals (n = 13). Panel D: Experimentally infected vaccinated animals (n = 14). Panel E: Naturally infected immunosuppressed control animals (n = 6). Panel F: Naturally infected immunosuppressed vaccinated animals (n = 9). Panel G: Experimentally infected immunosuppressed control animals (n = 6). Panel H: Experimentally infected immunosuppressed vaccinated animals (n = 6). The red line depicts the linear correlation between the neutralizing and the anti-L1 titer for the 113 sera (Fig. 2B). n: indicates the number of animals. (TIF) [file ppat.1003924.s004.tif]

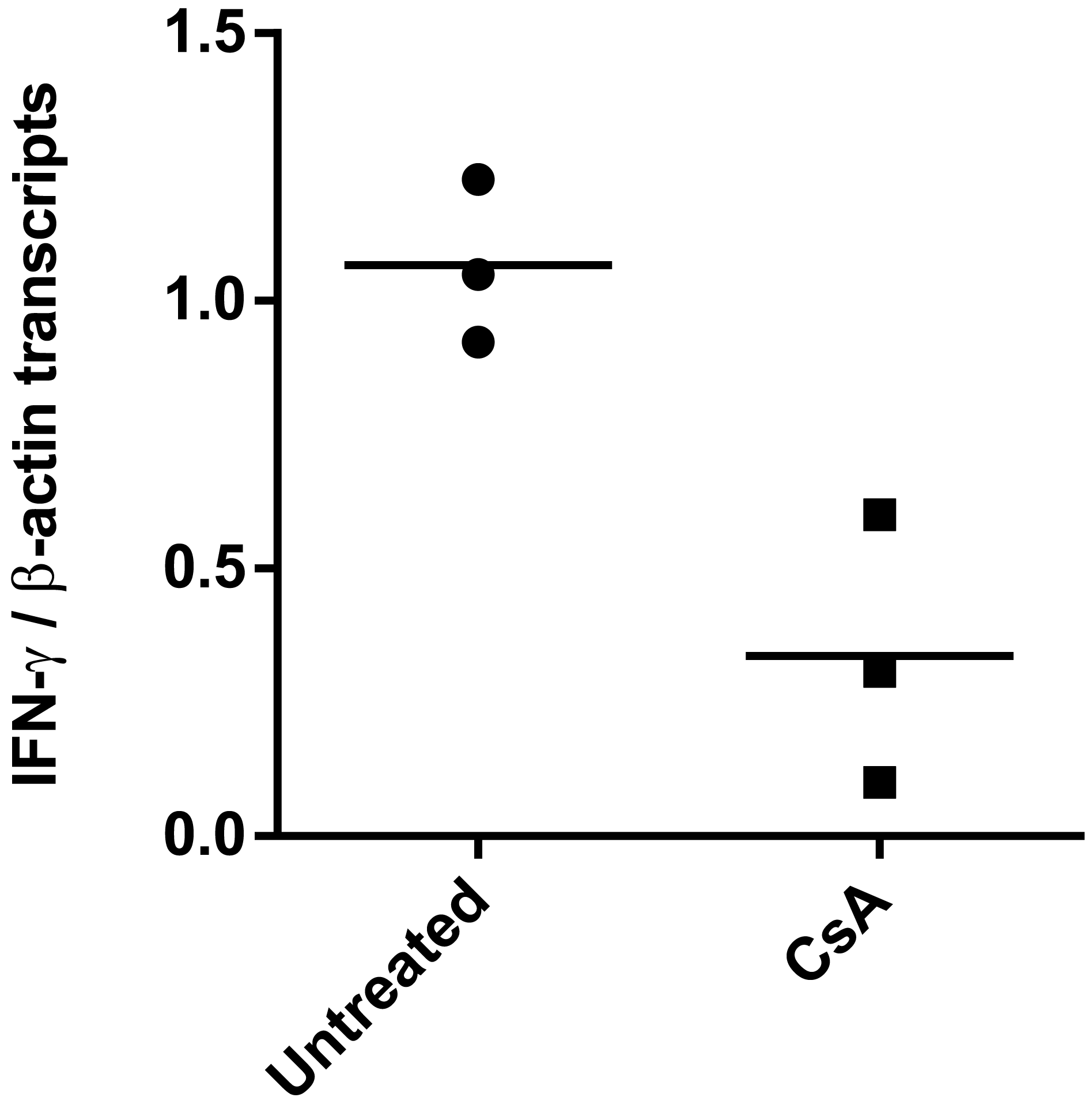

Supplement: Figure S5 — Effect of CsA feeding on the expression of IFN-γ. Spleens were dissected from Mastomys following a normal diet (•) or receiving food containing a low concentration (125 mg/kg) of CsA (▪). Splenocytes were isolated as described elsewhere (3) and stimulated with 2 µg/mL Concanavalin A (Sigma) for four days. RNA was extracted by using the RNeasy Kit (QIAGEN) according to the manufacturer's instructions. To eliminate all traces of viral DNA in order to avoid false positive signals by RT-PCR, the RNA was additionally treated with DNase I (QIAGEN). Reverse transcription was performed with the reverse transcriptase SuperScript II (Invitrogen) according to the manual. Quantification of IFN-γ transcripts was performed with the iTaq Universal SYBR Green Supermix (Bio-Rad), following the manufacturer instructions. Detection was done with the CFX96 real time PCR detection system (Bio-Rad). Primers were specifically designed to bind Mastomys IFN-γ transcripts (forward primer: 5′-CTGTTACTGCCAAGGCACAC-3′, reverse primer: 5′-CATCCTTTTGCCAGTTCCTC-3′). Data was normalized by the amount of β-actin transcripts in the same sample (forward primer: 5′-GAAGAGCTATGAGCTGCCTGAC-3′, reverse primer: 5′-GTTTCATGGATGCCACAGGA-3′). (TIF) [file ppat.1003924.s005.tif]
