# Supplementary material for: Protective Vaccination against Papillomavirus-Induced Skin Tumors under Immunocompetent and Immunosuppressive Conditions: A Preclinical Study Using a Natural Outbred Animal Model
Source: PLoS Pathog. 2014 Feb 20;10(2):e1003924. doi: 10.1371/journal.ppat.1003924 (PMC3930562; doi:10.1371/journal.ppat.1003924)
Supplement: References S1 — Supporting information references. (DOCX) [file ppat.1003924.s006.docx]

## Supporting References

1. Schäfer K, Neumann J, Waterboer T, Rösl F (2011) Serological markers for papillomavirus infection and skin tumour development in the rodent model Mastomys coucha. J Gen Virol 92:383-394.

2. Rubio I, et al. (2011) The N-terminal region of the human papillomavirus L2 protein contains overlapping binding sites for neutralizing, cross-neutralizing and non-neutralizing antibodies. Virology 409(2):348-359.

3. Kruisbeek AM (2001) Isolation of mouse mononuclear cells. Curr Protoc Immunol Chapter 3:Unit 3.1.
