# Supplementary material for: Protective Vaccination against Papillomavirus-Induced Skin Tumors under Immunocompetent and Immunosuppressive Conditions: A Preclinical Study Using a Natural Outbred Animal Model
Source: PLoS Pathog. 2014 Feb 20;10(2):e1003924. doi: 10.1371/journal.ppat.1003924 (PMC3930562; doi:10.1371/journal.ppat.1003924)
Supplement: Table S1 — Statistical analysis of anti-L1 antibody titers. A non-parametric Kruskal–Wallis analysis followed by a Dunn's post hoc multiple comparison test was performed to compare all groups in the study. Graphpad Prism was used for the analysis. (DOCX) [file ppat.1003924.s007.docx]

**Table S1: Statistical analysis of anti-L1 antibody titers**

|  | **Significant difference between unvaccinated and vaccinated group?** | | | |
| --- | --- | --- | --- | --- |
|  | **Naturally infected animals** | | **Experimentally infected animals** | |
|  | **Immunocompetent** | **Immunosuppressed** | **Immunocompetent** | **Immunosuppressed** |
| **1.8-mo old** | **No** | **No** | **No** | **No** |
| **3.2-mo old** | **Yes (p<0.0001)** | **Yes (p<0.0001)** | **Yes (p<0.0001)** | **Yes (p<0.0001)** |
| **5-mo old** | **Yes (p<0.01)** | **No** | **Yes (p<0.0001)** | **Yes (p<0.05)** |
| **7-mo old** | **No** | **No** | **No** | **No** |
| **9-mo old** | **No** | **No** | **No** | **No** |
| **11-mo old** | **No** | **No** | **No** | **No** |
| **13-mo old** | **No** | **No** | **No** | **No** |
| **17-mo old** | **No** | **No** | **No** | **No** |

A non-parametric Kruskal–Wallis analysis followed by a Dunn's post hoc multiple comparison test was performed to compare all groups in the study. Graphpad Prism was used for the analysis.
